# HSC-Explorer: A Curated Database for Hematopoietic Stem Cells

**DOI:** 10.1371/journal.pone.0070348

**Published:** 2013-07-30

**Authors:** Corinna Montrone, Konstantinos D. Kokkaliaris, Dirk Loeffler, Martin Lechner, Gabi Kastenmüller, Timm Schroeder, Andreas Ruepp

**Affiliations:** 1 Institute for Bioinformatics and Systems Biology (IBIS), Helmholtz Zentrum München - German Research Center for Environmental Health (GmbH), Neuherberg, Germany; 2 Stem Cell Dynamics Research Unit, Helmholtz Zentrum München - German Research Center for Environmental Health (GmbH), Neuherberg, Germany; Emory University, United States of America

## Abstract

HSC-Explorer (http://mips.helmholtz-muenchen.de/HSC/) is a publicly available, integrative database containing detailed information about the early steps of hematopoiesis. The resource aims at providing fast and easy access to relevant information, in particular to the complex network of interacting cell types and molecules, from the wealth of publications in the field through visualization interfaces. It provides structured information on more than 7000 experimentally validated interactions between molecules, bioprocesses and environmental factors. Information is manually derived by critical reading of the scientific literature from expert annotators. Hematopoiesis-relevant interactions are accompanied with context information such as model organisms and experimental methods for enabling assessment of reliability and relevance of experimental results. Usage of established vocabularies facilitates downstream bioinformatics applications and to convert the results into complex networks. Several predefined datasets (Selected topics) offer insights into stem cell behavior, the stem cell niche and signaling processes supporting hematopoietic stem cell maintenance. HSC-Explorer provides a versatile web-based resource for scientists entering the field of hematopoiesis enabling users to inspect the associated biological processes through interactive graphical presentation.

## Introduction

The term “Hematopoiesis” describes the life-long regeneration and repair of the blood system. All blood cells are ultimately generated from multipotent hematopoietic stem cells (HSCs) which are the only cell type capable of long-term (if not life-long) self-renewal (i.e. generation of daughter cells with HSC potential). According to the classical view of hematopoiesis, HSCs generate multipotent and committed progenitors which produce terminally differentiated cells. Characterized by a massive production rate (10^11–12^ blood cells per day in an adult human), hematopoiesis is tightly regulated by intrinsic mechanisms as well as extrinsic cues which balance various cellular behaviors, such as quiescence, self-renewal, differentiation, homing and migration.

In order to study these behaviors hematologists have been enriching hematopoietic stem cells for over 25 years using various purification strategies utilizing flow cytometry and functional in vitro and in vivo assays [1], [2]. While in the 1990’s the stem cell enrichment was dominated by the usage of three to four markers (cKIT, sca-1, CD34 and a mixture of several blood lineage specific markers) reaching purities of at least 20% [2], technical advances during the last decade made it possible to distinguish subpopulations with theoretically up to 17 markers [3]. The utilization of additional markers in recent years has led to the emergence of a variety of purification strategies yielding stem cell purities over 50% [4], [5]. Although some of these strategies are closely related, others utilize a completely different set of markers. If and to what extent the results of different purification strategies are comparable is unclear. A comparison of the gene expression profile between HSC populations purified using different enrichment protocols suggests that it might be limited [6].

In addition to that, the criteria for cells to be classified as HSCs keep changing every few years (i.e. length of repopulation/contribution upon transplantation). Terminologies like Long-Term (LT) and Short-Term (ST) HSCs are functionally defined (LT-HSCs repopulate mice longer than 16 weeks, ST-HSCs shorter than 12 weeks) and do not necessarily correlate with certain enrichment protocols. However, the use of such terms is not consistent throughout the literature. This inconsistency together with the intrinsic heterogeneity of the HSC compartment [7], [8] hampers proper interpretation and direct comparison of results from different publications. For this reason a comprehensive understanding of the current knowledge in the field requires the collection of the various experimental results, within a unified resource.

Currently, hematopoiesis-specific databases such as Hematopoietic Fingerprints [9], HemoPDB [10] or StemBase [11] collecting information about gene expression or transcriptional regulation in hematopoiesis are available. However, there is a demand for a resource that provides information about the interactions between cellular components and signaling processes characterizing the diverse stem cell subpopulations isolated so far and their stem cell-related functions also in context with the stem cell niche.

Here we present HSC-Explorer, a publicly available, manually curated, integrative database collecting literature-derived knowledge about the different hematopoietic stem cell subpopulations and their behavior in repopulation activity, self-renewal and quiescence and how these processes are regulated by intrinsic and extrinsic factors. The resource covers in particular the early steps of differentiation from the most primitive hematopoietic stem cells (HSC) to more differentiated multipotent progenitor cells (MPP) in adult mice. Multiple search options and an interactive graphical tool enable information retrieval of the manifold interrelations between factors and processes and their presentation as informative network structures.

## Results and Discussion

### Curation of Information from hematopoietic literature

**Figure 1:**
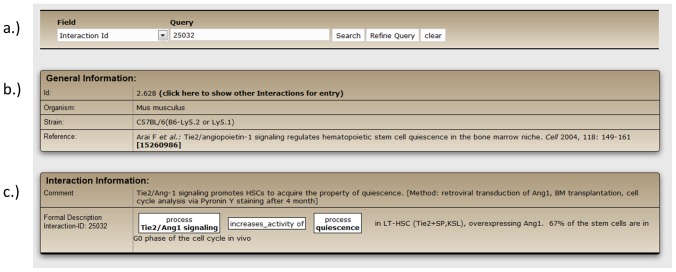
Detailed curation of an interaction in HSC-Explorer. Information about the interaction between ‘Tie2/Ang1 signaling’ and the term ‘quiescence’ consists of (a) ‘General information’ about the organism used in the experiment and reference, (b) textual information (‘Comment’) about the interaction and experimental procedure, and (c) structured information including in addition information about the hematopoietic cell-type.

The complete content of the database is generated by biocurators who manually extract hematopoiesis-specific and experimentally verified information from the scientific literature. Annotation is performed according to the procedures applied in our CIDeR database [12] and if required adapted to the peculiarities in hematopoiesis. Information in HSC-Explorer is described using three types of information (Figure 1): general information (Figure 1A), textual information (comment) (Figure 1B), and structured, machine-readable information (Figure 1C).

The general information (A) refers to the broader context of the experimental findings. This includes the literature reference, the organism used for the experiments and information about the organism strain, gender and age, if specified in the publication. The information about mouse strains is especially important for the purification of murine HSCs since some frequently used stem cell markers, including Thy-1 and Sca-1, are not conserved among mouse strains [5], [13]. Since most studies in the field of hematopoietic stem cells are performed with mice, the vast majority of HSC-Explorer (90%) consists of experimental results from mice. Experimentally verified biological information is shown as short comments in the textual information part of the annotation (B). If appropriate, it also includes supplemental details about the experimental results, for example, the amount of cells that are found in different stages of the cell cycle. In addition, a short description of the methods used for the experiment is presented to inform the user how the data are obtained. The structured information (C) translates the biological findings into very basic information given as the relation between two elements. Elements can be genes (respective proteins), metabolites, miRNAs but also cellular processes, tissues (respective cell types) or a stem cell sub-population characterized by the marker combination (immunophenotype) used for its purification. For the curation of the structured information we use established resources such as Entrez Gene [14], Gene Ontology [15] and CORUM [16] and the vocabularies used therein. Standardized information is a prerequisite for applications such as the construction of biological networks and the generation of diagrams or subsequent post processing of data using bioinformatics methods. In addition, we indicate whether the result is obtained from in vitro or in vivo experiments.

All data from HSC-Explorer can be downloaded as flat files or in SBML format (Systems Biology Markup Language), a free and open interchange XML format for computer models of biological processes [17]. Graphical outputs can be downloaded in JPEG format or GraphML format. The latter can be opened and edited via the graph editor yED (yWorks GmbH, Tübingen, Germany). For the future, we will work on expanding the database to ultimately cover all hematopoietic progenitor populations and generate novel ‘Selected topics’.

### Database Contents

#### The HSC sub-populations and their activities

One crucial question in hematopoietic stem cell biology is the identification of markers that distinguish primitive stem cells from more differentiated progenitors with reduced self-renewal and repopulation activity [18]. In the last years several highly enriched HSC populations have been identified, for example by introducing SLAM markers for HSC enrichment [5], or by considering HSC-specific functional properties such as higher capability for Hoechst dye efflux (side population SP) [1], [7] or by detecting low levels of Rhodamine 123 staining (Rho(lo)) [8], [19]. HSC-Explorer provides a comprehensive collection of these HSC subpopulations (Table 1), illustrating the heterogeneity of stem cell enrichment protocols published so far. If applicable, the potential of each HSC population for multilineage reconstitution is stated in our database. Since the term “long-term-HSC” (LT-HSC) is heterogeneously used throughout the literature, we classify stem cells not only with the term “LT-HSC” but also by linking them to the process “repopulation >16 weeks”. Interestingly, repopulation activity was measured for longer than 32 weeks in some studies [2], [20]–[22]. These stem cells are linked with the process “repopulation >32 weeks”, to emphasize that their repopulation activity is higher than the currently used standard of 16 weeks. More differentiated progenitor cells are linked with the term “repopulation <12 weeks”.

**Table 1:** Populations enriched for hematopoietic stem cells.

Reference	Publication date	Immunophenotype	Multilineagereconstitution	Self-renewal	quiescence	Single cell
2898810	Spangrude, et al., 1988	HSC (Thy1(lo)Sca1+Lin-)	<12 weeks	NA	yes	
1281687	Okada et al., 1992	HSC (KSL)	>16 weeks	NA	NA	
1346154	Uchida et al., 1992	HSC (Thy1(lo)Sca1+Lin-)	>16 weeks	NA	NA	
1371359	Ikuta et al., 1992	HSC (Thy1(lo)KSL)	>16 weeks	NA	NA	
8662508	Osawa et al., 1996	HSC (CD34-KSL)	>32 weeks	high	NA	yes
8666936	Goodell et al., 1996	HSC (SP,Sca1+Lin-)	>16 weeks	NA	yes	
11672547	Adolfsson, et al., 2001	HSC (CD135-KSL)	>16 weeks	high	NA	
14738767	Matsukaki et al.,2004	HSC (CD34-SP(Tip),KSL)	>16 weeks	NA	NA	
15989959	Kiel et al., 2005	HSC (CD150+CD48-CD41-KSL)	>16 weeks	high	NA	
		HSC (CD150+CD48-CD244-)	>16 weeks	high	NA	
		HSC (CD150+Thy1(lo)KSL)	>16 weeks	high	NA	
18371352	Dykstra et al., 2007	HSCalpha (CD45(mid)lin-Rho-SP)	>16 weeks	high	NA	yes
		HSCbeta (CD45(mid)lin-Rho-SP)	>16 weeks	high	NA	yes
		HSCgamma (CD45(mid)lin-Rho-SP)	>16 weeks	NO	NA	yes
		HSCdelta (CD45(mid)lin-Rho-SP)	>16 weeks	NO	NA	yes
18055867	Weksberg et al.,2008	HSC (CD150+SP,KSL)	>16 weeks	high	yes	
		HSC (CD150-SP,KSL)	>16 weeks	high	yes	
19062086	Wilson et al., 2008	dormant-HSC(LRC-HSC, CD34-CD150+CD48-CD135-KSL)	>16 weeks	high	yes	
19377048	Kent et al., 2009	HSC (CD150+CD45+CD201+CD48-)	>16 weeks	high	NA	yes
		HSC (CD150-CD45+CD201+CD48-)	>16 weeks	low	NA	yes
20207229	Challen et al., 1010	My-bi-HSC (lower SP,KSL)	>16 weeks	high	yes	
		Ly-bi-HSC (upper SP,KSL)	>16 weeks	low	yes	
20074534	Benveniste et al., 2010	LT-HSC (CD34(lo)CD135-Rho(lo)CD49b(lo)KSL)	>32 weeks	high	yes	
		IT-HSC (CD34(lo)CD135-Rho(lo)CD49b(hi)KSL)	>16 weeks	low	yes	
		ST-HSC (CD34(hi)CD135+Rho(hi)KSL)	<12 weeks	NO	NO	
20421392	Morita, et al., 2010	HSC (CD150(hi)CD34-KSL)	>16 weeks	high	NA	
		HSC (CD150(int)CD34-KSL)	>16 weeks	low	NA	
		HSC (CD150-CD34-KSL)	>16 weeks	NO	NA	

**Table 2:** Bone marrow stromal cell types (endosteal) known to contribute to the hematopoietic microenvironment.

	PMID	Author	Niche cell type	Niche factor	HSC counterpart	HSC sub-type	Signaling process	Role
**Endosteal niche**	18371409	Yoshihara et al., 2007	Osteoblast (ALP^+^Thpo^+^)	Thrombopoietin (Thpo)	Mpl	CD34^-^MPL^+^KSL	TPO/MPL signaling	HSC self-renewal/ quiescence
	15260986	Arai et al., 2004	Osteoblast (osteocalcin^+^Ang1^+^)	Angiopoietin (Ang1)	Tek (Tie2)	Tie2^+^SP KSL	Tie2/Ang1 signaling	HSC adhesion/ quiescence
	14574413	Calvi et al., 2003	Osteoblast (osteopontin^+^Jag1^+^)	Jagged 1 (Jag1)	Notch1	KSL	Notch signaling pathway	
	20887955	Kieslinger et al., 2010	Osteoblast (Ebf2^+^)	Early B cell factor 2 (Ebf2)		Sca1^+^	Wnt signaling pathway	
	21108988	Jung et al., 2011	Endosteum (Anexin2^+^)	Annexin 2 (Anxa2), Chemokine (C-X-C motif) ligand 12 (Cxcl12)		CD150^+^CD48^-^CD41^-^Lin^-^	CXCL12/CXCR4 signaling	HSC homing and CXCL12-driven cell migration
	22817897	Sugimura et al., 2012	Osteoblast (N-cadherin^+^)	Flamingo (Fmi)/Frizzled (Fz8)	Flamingo (Fmi)/Frizzled (Fz8)	LRC, CD135^-^KSL/ Fmi^+^Fz8^+^	Non-canonical Wnt signaling / Ca2+ intracellular signaling	HSC quiescence
	14574412	Zhang et al., 2003	Spindel shaped (N-cadherin^+^CD45^-^) osteoblastic cells	N-cadherin (Cdh2)	N-cadherin (Cdh2)	LRC/N-cad^+^CD45^+^KSL	BMP signalling	
	22118468	Yamazaki et al., 2011	Osteoblast (osteocalcin^+^)			CD150^+^CD48^-^CD41^-^Lin^-^		
	21653324	Mazzon et al., 2011	Primary MSC-enriched cell (Alcam^-^Sca1^+^)	Agrin (Agrn)	Alpha-dystroglycan (Dag1)	CD34^+^CD135^-^KSL	Ras signaling	HSC apoptosis
	21868569	Arcangeli et al., 2011	Stromal cell (JamB^+^)	Junction adhesion molecule 3 (JamC)	Junction adhesion molecule 2 (JamB)	CD150^+^CD48^-^CD41^-^Lin^-^		HSC adhesion
	21131587	Lymperi et al., 2011	Osteoclast			CD135^-^KSL		HSC quiescence
	19062086	Wilson et al., 2008	Endosteum			LRC, Kit^+^		HSC quiescence
	20703299	Méndez-Ferrer et al., 2010	MSC (Nestin^+^)			CD150^+^CD48^-^Lin^-^		HSC homing
	20713966	Winkler et al., 2010	Osteomac (F4/80^+^Ly6G^-^CD11b^+^)			CD150^+^CD48^-^KSL		HSC mobilization
	19516257	Naveiras et al., 2009	Adipocyte			CD135^-^KSL		HSC quiescence

In addition to their repopulation activity, hematopoietic stem cells are further characterized in HSC-Explorer by their proliferation state (quiescence) and their capacity to self-renew. The term “self-renewal” is only used in cases of reconstitution of secondary recipients.

In table 1 ‘milestone publications’ describing new markers, which highly improved the purification of HSC are summarized. The different attributes of these stem cell enriched populations concerning repopulation activity, self-renewal and quiescence are indicated. Whenever the authors performed single cell transplantation this is mentioned in the table as well. The graphical network of these data as it is obtained with a search in the database with the term ‘keypopulation’ is shown in the figure S1.

#### The stem cell niche

The concept of the hematopoietic stem cell niche has been introduced in 1978 by Schofield [23] when he demonstrated that the bone marrow stromal cells play an active role in the regulation of the stem cell fate. Progress in the purification of HSCs [5] and in live imaging techniques [24] enabled the identification of candidate niche cells, which are all listed in tables 2 and 3 together with the HSC sub-population described in the respective study. These tables also list the genes and the corresponding signaling processes responsible for the interplay between stromal and hematopoietic cells. In addition, HSC-Explorer provides information about cellular processes important for establishing the HSC niche like homing, cell migration, mobilization and cell adhesion.

**Table 3:** Bone marrow stromal cell types (vascular/endothelial) known to contribute to the hematopoietic microenvironment.

	PMID	Author	Niche cell type	Niche factor	HSC counterpart	HSC sub-type	Signaling process	Role
**Vascular niche**	19797522	Lewandowski et al., 2010	Vascular endothelial cell (Pecam1^+^)	Vascular cell adhesion molecule 1 (Vcam1)		CFSE-labeled CD34^-^KSL		
	15989959	Kiel et al., 2005	Sinusoidal endothelium (MECA-32^+^)			CD150^+^CD48^-^CD41^-^Lin^-^		
	17174120	Sugiyama et al., 2006	CAR cells (Cxcl12^+^)	C-X-C motif ligand 12 (Cxcl12)	C-X-C chemokine receptor type 4 (Cxcr4)	Sca1^+^cKit^+^/CD150^+^CD48^-^CD41^-^	CXCL12/CXCR4 signaling	HSC quiescence
	20207228	Butler et al., 2010	Sinusoidal endothelium (VE-cadherin^+^)	Jagged	Notch	TNR.GFP^+^	Notch signaling pathway	
	22118468	Yamazaki et al., 2011	Sinusoidal endothelium (VE-cadherin^+^)			CD150^+^CD48^-^CD41^-^Lin^-^		
	22281595	Ding et al., 2012	Sinusoidal endothelium	Stem cell factor (SCF,Kitl)	Kit oncogene (c-Kit)	CD150^+^CD48^-^Lin^-^		HSC maintenance
	22118468	Yamazaki et al., 2011	Non-myelinated Schwann cell (GFAP^+^)	Integrin beta 8 (Itgb8)	Tgf-beta receptor	CD150^+^CD48^-^CD41^-^Lin^-^	Tgf-beta/SMAD signaling	HSC quiescence
	20703299	Méndez-Ferrer et al., 2010	MSC (Nestin^+^)			CD150+CD48-Lin-		HSC homing
	22281595	Ding et al., 2012	Perivascular cell (Lepr^+^)	Stem cell factor (SCF,Kitl)	Kit oncogene (c-Kit)	CD150^+^CD48^-^Lin^-^		HSC maintenance
	22983360	Ludin et al., 2011	Monocytes-macrophage (alpha-SMA^+^)	Prostaglandin-endoperoxide synthase 2 (Ptgs2, Cox2), C-X-C motif ligand 12 (Cxcl12)	C-X-C chemokine receptor type 4 (Cxcr4)	CD150^+^CD48^-^CD41^-^	ROS signaling/CXCL12/CXCR4 signaling	HSC maintenance
	21282381	Chow et al., 2011	Macrophage (CD169^+^)+MSC (Nes^+^)	C-X-C motif ligand 12 (Cxcl12)	C-X-C chemokine receptor type 4 (Cxcr4)	CD135^-^KSL	CXCL12/CXCR4 signaling	HSC adhesion/mobilization
	21115812	de Graaf et al., 2010	Megakaryocyte	Thyroid peroxidase (Tpo)	Myeloproliferative leukemia virus oncogene (Mpl)	CD34^-^CD135^-^KSL	TPO/MPL signaling	

#### Curation of high-throughput data

Distinct HSC sub-populations are also characterized by their expression profiles. Several microarray studies have been performed to identify candidate genes involved in differentiation and self-renewal or quiescence [9], [25]. In total, the 3590 most significantly, differentially expressed genes identified in these large-scale analyses are mentioned and can be retrieved from the database with a search for “expression profile”. As already discussed by Vogel [26], expression profiles of HSC populations analyzed in different labs are almost completely different, even if the same purification procedures have been used for isolating the stem cells, indicating that the populations were heterogeneous [27]. However, the inclusion of various kinds of experimental data in HSC-Explorer allows combining genes which have been found by only large-scale expression analysis with experimentally verified data.

Therefore we compared the expression profiles, including microarray data and qPCR data, of four different HSC sub-populations (HSC (CD34-SP,KSL), HSC (Rho(lo)KSL), HSC (SP,KSL), HSC (Thy1(lo)CD135-KSL) ) with long-term repopulation activity. As shown in the graph on the homepage (Selected topic) the gene sets are quite different and only two genes, Procr and Rbp1, have been identified in all four subpopulations. Procr is a well-known surface antigen already used for the purification of hematopoietic stem cells. The retinol-binding protein Rbp1, an important factor in retinoic acid synthesis, is involved in granulopoiesis [28] and has recently discussed to be hypermethylated in glioma tumors, resulting in decreased expression of Rbp1 [29]. To our knowledge a role of Rbp1 in the differentiation of hematopoietc stem cells has so far not been shown and would be interesting to investigate.

### Search Options and Web Interface

A flexible web-based interface of HSC-Explorer allows both general and advanced searching of the database. By default all fields are searched. However, the user has the option to restrict the search to a specific category such as ‘Immunophenotype’, ‘Gene’, ‘Biological process’, ‘Tissue’, ‘Chemical compound’, ‘miRNA’, the PubMed identifier as well as the authors of articles. The category ‘Immunophenotype’ had to be introduced in this context as currently no single defined hematopoietic stem cell exists, instead, a variety of different HSC-sub-populations have been described in the literature so far. All these sub-types are characterized by a combination of markers used for their purification, the so-called immunophenotype (for example HSC CD150^+^CD48^-^KSL [cKit^+^Sca1^+^Lin^−^]). Genes are described by their official Entrez Gene name [14] (e.g. Slamf1). For convenient retrieval of required information respective synonyms from Entrez Gene and KEGG [30] are also included for searches. The category ‘Tissue’ summarizes all the different stromal cell types known to establish the stem cell niche. Stem-cell-specific processes such as ‘repopulation activity’, ‘self-renewal activity’ or ‘quiescence’ are summarized in the search field ‘Biological process’.

Search results can be improved further with iterative searches by using the ‘Refine Query’ option. Results of the new search term are combined with the first search term by using one of the three Boolean operators ‘AND’ ‘OR’ and ‘NOT’. The search results are listed as tables that can either be linked to the complete annotated information or can be graphically visualized with a tool that dynamically generates an interaction network. Graphs are interactive and provide several options for retrieval of information or exploration of interaction networks. While moving the mouse cursor over the edges, pop-up windows with information about the respective interaction appear. Another useful function is the extension of the graph. By double-clicking on a node of interest all additional relations linked to the node are shown. Further functionalities about the database and the graph tool are explained in online help documentations and two tutorial movies.

In addition to the ability to generate user-defined graphs, HSC-Explorer provides several predefined graphs (Selected topics) on the homepage displaying, for example, proteins known to influence the proliferation state (quiescence) or the self-renewal activity of a stem cell. Short summaries of signaling processes known to affect hematopoiesis, such as Cxcl12/Cxcr4 signaling, Ang1/Tie2 or Notch signaling are presented as well. A graph, showing an overview about niche-related data, including stem cell sub-populations, cellular components of the bone marrow niche, extrinsic or intrinsic factors and signaling processes necessary for establishing the niche is provided. A small outline thereof, displaying only the co-localization of HSCs to non-hematopoietic tissue is given as a selected topic as well.

### Application Examples

An objective of HSC-Explorer is to present complex experimental results relevant for murine hematopoiesis as an intuitive graphical output. The following examples illustrate the capability of HSC-Explorer to display complex data in a comprehensive and easy to follow manner.

#### 1. The role of Cxcl12 signaling in homing and engraftment of hematopoietic stem cells

**Figure 2:**
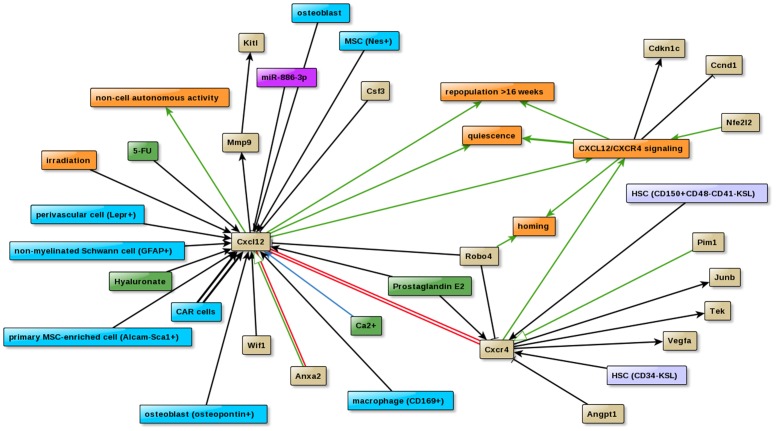
Graphical presentation of the ‘Cxcl12/Cxcr4 signaling’ network. The graph illustrates the process of Cxcl12/Cxcr4 signaling (described in the text) based on the interactions between heterogeneous factors such as proteins (beige), bioprocesses (orange), chemical compounds (green), niche cell types (cyan), hematopoietic stem/progenitor cells (light blue) and a microRNA (purple).

The chemokine Cxcl12 plays a key role during ontogeny of the hematopoietic system. A graphical visualization of the Cxcl12 interactions in HSC-Explorer clearly shows that Cxcl12 is mainly expressed in non-hematopoietic tissue like osteoblasts [31] or CAR cells [32]. Extension of the network with the Cxcr4 interactions reveals the following results (Fig. 2): (i) The chemokine receptor Cxcr4 is mainly expressed in LT-HSCs (e.g. HSC (CD34^-^KSL) or HSC (CD150^+^CD48^-^CD41^-^KSL). Cxcl12/Cxcr4 signaling not only plays a pivotal role in the regulation of HSC homing [discussed in [33]] and LT-repopulation activity of stem cells [34], but is also involved in the maintenance of quiescence [34], probably by increasing the expression level of the cell cycle inhibitor Cdkn1c (p57-kip2) [35] or by down-regulating of the expression of other cell cycle regulators such as cyclin D1 (Ccnd1) [35]. (ii) The graph shows that depletion of Cxcr4 results in an down-regulation of HSC regulators like Tek (Tie2), Vegfa and Junb in HSC (CD34^-^KSL) [34]. (iii) Addition of Angiopoietin (Angpt1) inhibits Cxcr4 expression in vitro [36] whereas Wif1, a known Wnt signaling inhibitor increases the expression of Cxcl12 [37]. (iv) Not only genes but also miRNAs are involved in the regulation of Cxcl12 expression. Transfection of miR-886-3p into a Cxcl12^+^ stromal cell results in a down-regulation of Cxcl12 [38]. (v) Furthermore, the graph reveals the cooperation of Robo4 with Cxcr4 in the homing process. Loss of Robo4 is compensated by up-regulation of Cxcr4 expression [39]. (vi) High levels of Cxcl12, e.g. induced by 5-FU treatment or irradiation, are known to elevate MMP9 levels [40] which results in high amounts of soluble KitL, a factor important for cell mobilization [41].

#### 2. Graphical visualization of the opposing effects on hematopoiesis induced by the polycomb repressive complex PRC1

**Figure 3:**
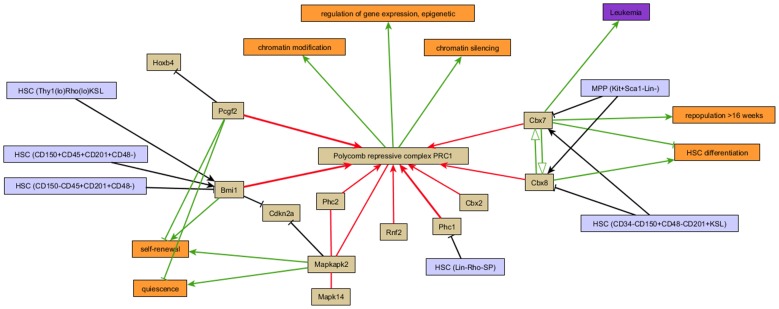
Influence of the subunit composition of PRC1 on the fate of hematopoietic stem cells. The subunit composition of the polycomb repressive complex (PRC1) is responsible for the balance between self-renewal and differentiation of hematopoietic stem cells. Cbx7 and Cbx8 compete for integration into the PRC1 complex. While Cbx7 inhibits HSC differentiation and induces self-renewal, Cbx8 has the opposite effect. Interaction types indicate ‘is part of’ (red arrow), ‘increase activity’ (green arrow), ‘affects activity’ (open green arrow), ‘increase expression’ (black arrow) and ‘interacts’ (red line). T-bar arrows indicate inhibitory behaviour.

Fine control of gene expression by modulating the chromatin structure is a widely used mechanism in eukaryotes to regulate cell development. The multisubunit polycomb repressive complex PRC1 catalyzes histone modifications and has been implicated in the maintenance of hematopoietic stem cells. Figure 3 illustrates that the subunit composition of PRC1 is responsible for the balance between self-renewal and differentiation of hematopoietic stem cells. Cbx7 is highest expressed in primitive stem cells, whereas the protein levels of Cbx8 increases during lineage commitment [42]. Both Cbx genes compete for integration into the PRC1 complex and have different effects on stem cell activity. While Cbx7 inhibits HSC differentiation and induces self-renewal, Cbx8 has the opposite effect. In addition it is known that the polycomb-subunit Bmi1 has a positive effect on stemness of adult hematopoietic stem cells [43], whereas the Bmi1 paralogue Pcgf2 (Mel18) negatively regulates the self-renewal activity [44]. But not only the composition of the complex is decisive. PRC1 is also regulated by external MAKPK kinases such as Mapkap2 (MK2) to improve the balancing between HSC self-renewal and differentiation [45].

In conclusion, HSC-Explorer provides a publicly available integrative resource in the field of hematopoiesis. HSC-Explorer has been developed to present biological findings in form of a comprehensive and intuitive graphical network, which will enable scientists to explore hematopoiesis in a more systems-oriented approach.

## Supporting Information

Figure S1 **Graphical presentation of** **table 1** **showing populations enriched for hematopoietic stem cells and their behavior in repopulation activity, self-renewal and quiescence.**
(TIF)

## References

[1] Goodell MA, Brose K, Paradis G, Conner AS, Mulligan RC (1996) Isolation and functional properties of murine hematopoietic stem cells that are replicating in vivo. J Exp Med 183: 1797–1806. 8666936 10.1084/jem.183.4.1797 PMC2192511

[2] Osawa M, Hanada K, Hamada H, Nakauchi H (1996) Long-term lymphohematopoietic reconstitution by a single CD34-low/negative hematopoietic stem cell. Science 273: 242–245. 8662508 10.1126/science.273.5272.242

[3] Perfetto SP, Chattopadhyay PK, Roederer M (2004) Seventeen-colour flow cytometry: unravelling the immune system. Nat Rev Immunol 4: 648–655. 15286731 10.1038/nri1416

[4] Kent DG, Copley MR, Benz C, Wohrer S, Dykstra BJ, et al (2009) Prospective isolation and molecular characterization of hematopoietic stem cells with durable self-renewal potential. Blood 113: 6342–6350. 19377048 10.1182/blood-2008-12-192054

[5] Kiel MJ, Yilmaz OH, Iwashita T, Yilmaz OH, Terhorst C, et al (2005) SLAM family receptors distinguish hematopoietic stem and progenitor cells and reveal endothelial niches for stem cells. Cell 121: 1109–1121. 15989959 10.1016/j.cell.2005.05.026

[6] Forsberg EC, Passegue E, Prohaska SS, Wagers AJ, Koeva M, et al (2010) Molecular signatures of quiescent, mobilized and leukemia-initiating hematopoietic stem cells. PLoS One 5: e8785. 20098702 10.1371/journal.pone.0008785 PMC2808351

[7] Challen GA, Boles NC, Chambers SM, Goodell MA (2010) Distinct hematopoietic stem cell subtypes are differentially regulated by TGF-beta1. Cell Stem Cell 6: 265–278. 20207229 10.1016/j.stem.2010.02.002 PMC2837284

[8] Dykstra B, Kent D, Bowie M, McCaffrey L, Hamilton M, et al (2007) Long-term propagation of distinct hematopoietic differentiation programs in vivo. Cell Stem Cell 1: 218–229. 18371352 10.1016/j.stem.2007.05.015

[9] Chambers SM, Boles NC, Lin KY, Tierney MP, Bowman TV, et al (2007) Hematopoietic fingerprints: an expression database of stem cells and their progeny. Cell Stem Cell 1: 578–591. 18371395 10.1016/j.stem.2007.10.003 PMC2475548

[10] Pohar TT, Sun H, Davuluri RV (2004) HemoPDB: Hematopoiesis Promoter Database, an information resource of transcriptional regulation in blood cell development. Nucleic Acids Res 32: D86–D90. 14681365 10.1093/nar/gkh056 PMC308790

[11] Sandie R, Palidwor GA, Huska MR, Porter CJ, Krzyzanowski PM, et al (2009) Recent developments in StemBase: a tool to study gene expression in human and murine stem cells. BMC Res Notes 2: 39. 19284540 10.1186/1756-0500-2-39 PMC2660910

[12] Lechner M, Hohn V, Brauner B, Dunger I, Fobo G, et al (2012) CIDeR: multifactorial interaction networks in human diseases. Genome Biol 13: R62. 22809392 10.1186/gb-2012-13-7-r62 PMC3491383

[13] Spangrude GJ, Brooks DM (1992) Phenotypic analysis of mouse hematopoietic stem cells shows a Thy-1-negative subset. Blood 80: 1957–1964. 1356513

[14] Maglott D, Ostell J, Pruitt KD, Tatusova T (2011) Entrez Gene: gene-centered information at NCBI. Nucleic Acids Res 39: D52–D57. 21115458 10.1093/nar/gkq1237 PMC3013746

[15] Ashburner M, Ball CA, Blake JA, Botstein D, Butler H, et al (2000) Gene ontology: tool for the unification of biology. The Gene Ontology Consortium. Nat Genet 25: 25–29. 10802651 10.1038/75556 PMC3037419

[16] Ruepp A, Waegele B, Lechner M, Brauner B, Dunger-Kaltenbach I, et al (2010) CORUM: the comprehensive resource of mammalian protein complexes–2009. Nucleic Acids Res 38: D497–D501. 19884131 10.1093/nar/gkp914 PMC2808912

[17] Hucka M, Finney A, Sauro HM, Bolouri H, Doyle JC, et al (2003) The systems biology markup language (SBML): a medium for representation and exchange of biochemical network models. Bioinformatics 19: 524–531. 12611808 10.1093/bioinformatics/btg015

[18] Spangrude GJ, Heimfeld S, Weissman IL (1988) Purification and characterization of mouse hematopoietic stem cells. Science 241: 58–62. 2898810 10.1126/science.2898810

[19] Ivanova NB, Dimos JT, Schaniel C, Hackney JA, Moore KA, et al (2002) A stem cell molecular signature. Science 298: 601–604. 12228721 10.1126/science.1073823

[20] Benveniste P, Frelin C, Janmohamed S, Barbara M, Herrington R, et al (2010) Intermediate-term hematopoietic stem cells with extended but time-limited reconstitution potential. Cell Stem Cell 6: 48–58. 20074534 10.1016/j.stem.2009.11.014

[21] Muller-Sieburg CE, Cho RH, Thoman M, Adkins B, Sieburg HB (2002) Deterministic regulation of hematopoietic stem cell self-renewal and differentiation. Blood 100: 1302–1309. 12149211

[22] Muller-Sieburg CE, Cho RH, Karlsson L, Huang JF, Sieburg HB (2004) Myeloid-biased hematopoietic stem cells have extensive self-renewal capacity but generate diminished lymphoid progeny with impaired IL-7 responsiveness. Blood 103: 4111–4118. 14976059 10.1182/blood-2003-10-3448

[23] Schofield R (1978) The relationship between the spleen colony-forming cell and the haemopoietic stem cell. Blood Cells 4: 7–25. 747780

[24] Xie Y, Yin T, Wiegraebe W, He XC, Miller D, et al (2009) Detection of functional haematopoietic stem cell niche using real-time imaging. Nature 457: 97–101. 19052548 10.1038/nature07639

[25] Ivanova NB, Dimos JT, Schaniel C, Hackney JA, Moore KA, et al (2002) A stem cell molecular signature. Science 298: 601–604. 12228721 10.1126/science.1073823

[26] Vogel G (2003) Stem cells. ‘Stemness’ genes still elusive. Science 302: 371. 14563977 10.1126/science.302.5644.371a

[27] Seita J, Sahoo D, Rossi DJ, Bhattacharya D, Serwold T, et al (2012) Gene Expression Commons: an open platform for absolute gene expression profiling. PLoS One 7: e40321. 22815738 10.1371/journal.pone.0040321 PMC3399844

[28] Kastner P, Lawrence HJ, Waltzinger C, Ghyselinck NB, Chambon P, et al (2001) Positive and negative regulation of granulopoiesis by endogenous RARalpha. Blood 97: 1314–1320. 11222375 10.1182/blood.v97.5.1314

[29] Chou AP, Chowdhury R, Li S, Chen W, Kim AJ, et al (2012) Identification of retinol binding protein 1 promoter hypermethylation in isocitrate dehydrogenase 1 and 2 mutant gliomas. J Natl Cancer Inst 104: 1458–1469. 22945948 10.1093/jnci/djs357 PMC3529615

[30] Kanehisa M, Goto S, Furumichi M, Tanabe M, Hirakawa M (2010) KEGG for representation and analysis of molecular networks involving diseases and drugs. Nucleic Acids Res 38: D355–D360. 19880382 10.1093/nar/gkp896 PMC2808910

[31] Calvi LM, Adams GB, Weibrecht KW, Weber JM, Olson DP, et al (2003) Osteoblastic cells regulate the haematopoietic stem cell niche. Nature 425: 841–846. 14574413 10.1038/nature02040

[32] Omatsu Y, Sugiyama T, Kohara H, Kondoh G, Fujii N, et al (2010) The essential functions of adipo-osteogenic progenitors as the hematopoietic stem and progenitor cell niche. Immunity 33: 387–399. 20850355 10.1016/j.immuni.2010.08.017

[33] Sharma M, Afrin F, Satija N, Tripathi RP, Gangenahalli GU (2011) Stromal-derived factor-1/CXCR4 signaling: indispensable role in homing and engraftment of hematopoietic stem cells in bone marrow. Stem Cells Dev 20: 933–946. 21186999 10.1089/scd.2010.0263

[34] Sugiyama T, Kohara H, Noda M, Nagasawa T (2006) Maintenance of the hematopoietic stem cell pool by CXCL12-CXCR4 chemokine signaling in bone marrow stromal cell niches. Immunity 25: 977–988. 17174120 10.1016/j.immuni.2006.10.016

[35] Nie Y, Han YC, Zou YR (2008) CXCR4 is required for the quiescence of primitive hematopoietic cells. J Exp Med 205: 777–783. 18378795 10.1084/jem.20072513 PMC2292218

[36] Arai F, Hirao A, Ohmura M, Sato H, Matsuoka S, et al (2004) Tie2/angiopoietin-1 signaling regulates hematopoietic stem cell quiescence in the bone marrow niche. Cell 118: 149–161. 15260986 10.1016/j.cell.2004.07.004

[37] Schaniel C, Sirabella D, Qiu J, Niu X, Lemischka IR, et al (2011) Wnt-inhibitory factor 1 dysregulation of the bone marrow niche exhausts hematopoietic stem cells. Blood 118: 2420–2429. 21652676 10.1182/blood-2010-09-305664 PMC3167356

[38] Pillai MM, Yang X, Balakrishnan I, Bemis L, Torok-Storb B (2010) MiR-886–3p down regulates CXCL12 (SDF1) expression in human marrow stromal cells. PLoS One 5: e14304. 21179442 10.1371/journal.pone.0014304 PMC3001477

[39] Smith-Berdan S, Nguyen A, Hassanein D, Zimmer M, Ugarte F, et al (2011) Robo4 cooperates with CXCR4 to specify hematopoietic stem cell localization to bone marrow niches. Cell Stem Cell 8: 72–83. 21211783 10.1016/j.stem.2010.11.030 PMC3625377

[40] Heissig B, Hattori K, Dias S, Friedrich M, Ferris B, et al (2002) Recruitment of stem and progenitor cells from the bone marrow niche requires MMP-9 mediated release of kit-ligand. Cell 109: 625–637. 12062105 10.1016/s0092-8674(02)00754-7 PMC2826110

[41] Ikuta K, Weissman IL (1992) Evidence that hematopoietic stem cells express mouse c-kit but do not depend on steel factor for their generation. Proc Natl Acad Sci U S A 89: 1502–1506. 1371359 10.1073/pnas.89.4.1502 PMC48479

[42] Klauke K, Radulovic V, Broekhuis M, Weersing E, Zwart E, et al (2013) Polycomb Cbx family members mediate the balance between haematopoietic stem cell self-renewal and differentiation. Nat Cell Biol 15: 353–362. 23502315 10.1038/ncb2701

[43] Iwama A, Oguro H, Negishi M, Kato Y, Morita Y, et al (2004) Enhanced self-renewal of hematopoietic stem cells mediated by the polycomb gene product Bmi-1. Immunity 21: 843–851. 15589172 10.1016/j.immuni.2004.11.004

[44] Kajiume T, Ninomiya Y, Ishihara H, Kanno R, Kanno M (2004) Polycomb group gene mel-18 modulates the self-renewal activity and cell cycle status of hematopoietic stem cells. Exp Hematol 32: 571–578. 15183898 10.1016/j.exphem.2004.03.001

[45] Schwermann J, Rathinam C, Schubert M, Schumacher S, Noyan F, et al (2009) MAPKAP kinase MK2 maintains self-renewal capacity of haematopoietic stem cells. EMBO J 28: 1392–1406. 19369945 10.1038/emboj.2009.100 PMC2688528

