# Supplementary figures and images for: HSC-Explorer: A Curated Database for Hematopoietic Stem Cells
Source: PLoS One. 2013 Jul 30;8(7):e70348. doi: 10.1371/journal.pone.0070348 (PMC3728102; doi:10.1371/journal.pone.0070348)

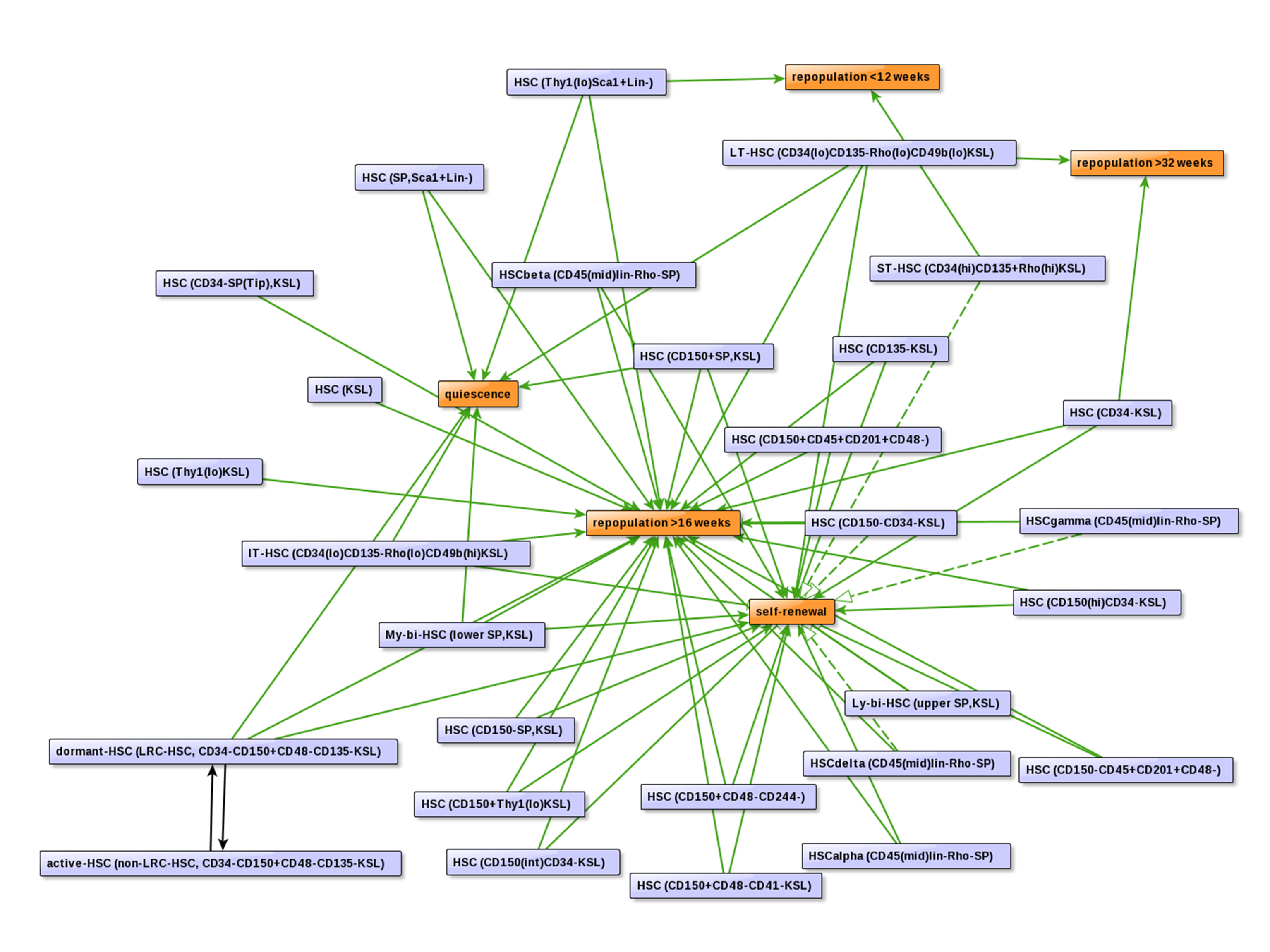

Supplement: Figure S1 — Graphical presentation of table 1 showing populations enriched for hematopoietic stem cells and their behavior in repopulation activity, self-renewal and quiescence. (TIF) [file pone.0070348.s001.tif]
